# Efficacy and safety of levetiracetam in preventing postoperative seizures in adult patients with brain tumors: a meta-analysis

**DOI:** 10.3389/fneur.2025.1543905

**Published:** 2025-03-07

**Authors:** Yongyi Zhang, Haoming Li, Bin Zhang, Junchen Zhang, Chengde Li

**Affiliations:** ^1^School of Pharmacy, Shandong Second Medical University, Weifang, China; ^2^Clinical Medicine School, Shandong Second Medical University, Weifang, China; ^3^Department of Neurosurgery, Affiliated Hospital of Jining Medical University, Jining, China

**Keywords:** brain tumor, levetiracetam, meta-analysis, prophylaxis, seizure

## Abstract

**Objective:**

Seizures are one of the most common symptoms in patients with brain tumor. The efficacy of prophylactic antiepileptic agents in reducing postoperative seizures in patients with brain tumor remains disputed. We conducted this meta-analysis to evaluate the efficacy and safety of levetiracetam in preventing seizures in adult patients with brain tumor.

**Review methods:**

We gathered studies comparing the effectiveness of levetiracetam with other antiepileptic drugs in preventing postoperative seizures in individuals with brain tumor from 2008 to 2023. We used the search terms levetiracetam, brain tumor, prevention, and seizures to retrieve relevant studies from the Pubmed, Medline, EMBASE, China National Knowledge Infrastructure, and Wanfang databases. The meta-analysis was conducted using RevMav 5.3 software.

**Results:**

After the literature search and screening, nine English-language studies involving a total of 2,433 patients were analyzed. The meta-analysis revealed that levetiracetam had higher efficacy for preventing overall seizures than the control intervention (odds ratio [OR] 0.56, 95% confidence interval [CI] 0.44–0.71, *p* < 0.00001). Subgroup analyses revealed that the efficacy of levetiracetam was superior to that of sodium valproate (OR 0.53, 95% CI 0.39–0.72, *p* < 0.0001) and phenytoin sodium (OR 0.35, 95% CI 0.19–0.62, *p* = 0.0004). No statistically significant difference in the efficacy of early seizure prophylaxis (OR 0.55, 95% CI 0.15–2.04, *p* = 0.37) was observed. The subgroup analysis revealed that the efficacy of levetiracetam for preventing early seizures was better than that of phenytion sodium (OR 0.13, 95% CI 0.03–0.56, *p* = 0.006). No statistically significant difference was noted in the preventive efficacy against late seizures (OR 0.75, 95% CI 0.27–2.03, *p* = 0.57). The incidence of adverse drug reactions was lower in the levetiracetam group than in the control group (OR 0.18, 95% CI 0.05–0.64, *p* = 0.008). Further subgroup analyses revealed that the incidence of adverse drug reactions in the levetiracetam group was lower than that in the phenytion sodium group (OR 0.06, 95% CI 0.02–0.21, *p* < 0.001).

**Conclusion:**

Prophylactic levetiracetam decreases the frequency of postoperative seizures, particularly early postoperative seizures, in individuals with brain tumor, with superior effectiveness to phenytion sodium and sodium valproate. In addition, levetiracetam induced only minor adverse effects, with a lower occurrence rate of adverse reactions than phenytion sodium and valproate. Nevertheless, a potential for bias exists. Due to the limited number of high-quality randomized controlled trials included in this meta-analysis, prospective, multicenter, ethnically diverse, high-quality studies on levetiracetam are essential to determine the efficacy of preventive levetiracetam in managing postoperative seizures.

**Systematic review registration:**

https://inplasy.com/inplasy-2023-6-0091/

## 1 Introduction

Epilepsy is one of the most common symptoms in patients with brain tumor. Approximately 20–45% of patients with brain tumor experience seizures, which have a detrimental impact on the postoperative clinical course, prognosis, hospitalization costs, and rehabilitationc (1, 2). Therefore, effectively managing seizures during the perioperative period is crucial. Surgery to remove the tumor and a combination of medications are commonly used to control seizures. A recent survey from the American Association of Neurological Surgeons revealed that approximately 63% of neurosurgeons routinely prescribe levetiracetam as a prophylactic Antiseizure medications (ASMs) to patients undergoing surgery for supratentorial brain tumor without a history of epilepsy (3). However, the prophylactic use of ASMs is a subject of debate, with the American Academy of Neurology advising against their use in patients with brain tumor. The efficacy of prophylactic ASMs in reducing postoperative seizures in patients with brain tumor remains disputed (4, 5). Furthermore, there is a lack of consensus on the optimal dose, duration, and method of administering levetiracetam prophylaxis (3, 4, 6).

Previous reviews, meta-analyses, and studies in this field have primarily concentrated on traditional antiepileptic drugs, such as sodium valproate and phenytoin sodium (PHT). In contrast, there is a scarcity of studies and meta-analyses focusing on newer ASMs, such as levetiracetam (5, 7). Levetiracetam gained approval from the United States Food and Drug Administration in 2006. Research indicates that newer ASMs, including levetiracetam, exhibit superior efficacy and reduced side effects compared with traditional options. However, there is a lack of high-quality randomized controlled trials (RCTs) assessing the effectiveness of newer ASMs in preventing seizures in individuals with brain tumor (4, 8). Furthermore, limited evidence exists on the use of levetiracetam as a standalone agent for preventing perioperative seizures in patients with brain tumor (3, 6).

We conducted this meta-analysis of relevant literature published between 2008 and 2023 to systematically evaluate the preventive impact of levetiracetam on seizures and its side effects in patients with brain tumor (4, 8). The aim of the study was to offer valuable insights to inform clinical treatment guidelines and consensus.

## 2 Methods

### 2.1 Registration protocol

This meta-analysis was performed to evaluate the effectiveness and safety of preventive levetiracetam in individuals with brain tumor. During the review process, adherence to the Preferred Reporting Items for Systematic reviews and Meta-Analyses (PRISMA) statement guidelines were followed. Before commencing the review, a protocol was established and registered under number INPLASY202360091 and DOI: 10.37766/inplasy2023.6.0091.

### 2.2 Study inclusion and exclusion criteria

The inclusion criteria were (1) type of study: controlled studies of levetiracetam for preventing seizures in patients with brain tumor. As far as possible, RCTs were selected. All study types (including RCTs, non-RCTs, prospective cohort studies, and retrospective studies) were eligible for inclusion; (2) study subjects: patients with brain tumor aged ≥18 years who had undergone craniotomy resection or biopsy surgery and were administered levetiracetam to prevent seizures during the perioperative period; (3) interventions: levetiracetam in the experimental group and other ASMs or no ASMs in the control group; and (4) outcome indicators: number of seizures in the levetiracetam combination control group, including the number of early seizures (within 1 week of surgery) and the number of late seizures (after 1 week of surgery), adverse drug reactions, diseases, and death rates.

The exclusion criteria were (1) study subjects: patients with craniotomy for diseases other than brain tumors, aged <18 years, pregnancy, breastfeeding, severe complications (including renal failure and hepatic failure); (2) studies on combined ASMs; and (3) studies that were repetitively published, of poor quality, or for which statistical data could not be extracted.

### 2.3 Search strategy

Different search strategies were developed for different databases according to the search strategy of the Cochrane Collaboration Network. All relevant literature published from January 2008 to January 2023 in the PubMed, Medline, EMBASE, China National Knowledge Infrastructure, and Wanfang databases were searched. The Chinese search terms included levetiracetam, brain tumor, seizure, and prevention. The English search terms included brain tumor, levetiracetam, prophylaxis, and seizures.

### 2.4 Quality assessment

The Newcastle-Ottawa Scale (NOS) was used to evaluate the quality of the literature. Two investigators independently evaluated the quality and extracted data from each study that met the inclusion criteria. In case of disagreement, the decision was made by the research team after collective discussion. The quality evaluation criteria were (1) the method of selecting the case-mix control group (maximum score of 4 points); (2) the comparability of the case and control groups (maximum score of 2 points); and (3) outcome evaluation (maximum score of 3 points). The maximum total score was 9, and ≥ 5 points was considered high-quality literature.

### 2.5 Extraction of information

Duplicate publications were first excluded, followed by irrelevant literature, which was identified by screening the titles and abstracts. Finally, the full text was read, and the studies that met the eligibility criteria were selected. Two trained evaluators independently conducted this procedure, with the judgment of a third evaluator in case of disagreement. Data extraction included two main areas: trial design and information related to seizure prevention. Comparisons of the effects of levetiracetam were examined in four main areas: total number of seizures, early seizures, late seizures, and adverse drug reactions.

### 2.6 Statistical analysis

The analysis of meta was conducted using the software RevMan 5.3. Utilizing the X^2^ test, an analysis of heterogeneity was performed. When *P* is >0.1, the studies exhibited good homogeneity, leading to the implementation of meta-analysis through the fixed effects model. Conversely, if *P* was <0.1, the variance was deemed statistically significant, indicating heterogeneity, with I^2^ computed to quantify the extent. If I^2^ is ≤50%, heterogeneity was considered acceptable; however, if I^2^ > 50%, it was classified as extensive heterogeneity. Subsequently, a meta-analysis was undertaken utilizing the random effects model, alongside a random regression or sensitivity analysis, to identify the underlying reasons for heterogeneity. Depending on the specific circumstances, either meta-regression, subgroup analysis, or sensitivity analysis was conducted to pinpoint the source of heterogeneity. Upon completion, the combined odds ratio (OR), relative risk ratio (RR), or weighted mean difference, along with a 95% confidence interval (CI), were computed. These outcomes were visually represented using forest plots, while the presence of publication bias was determined using funnel plots.

## 3 Results

### 3.1 Literature search results

**Figure 1:**
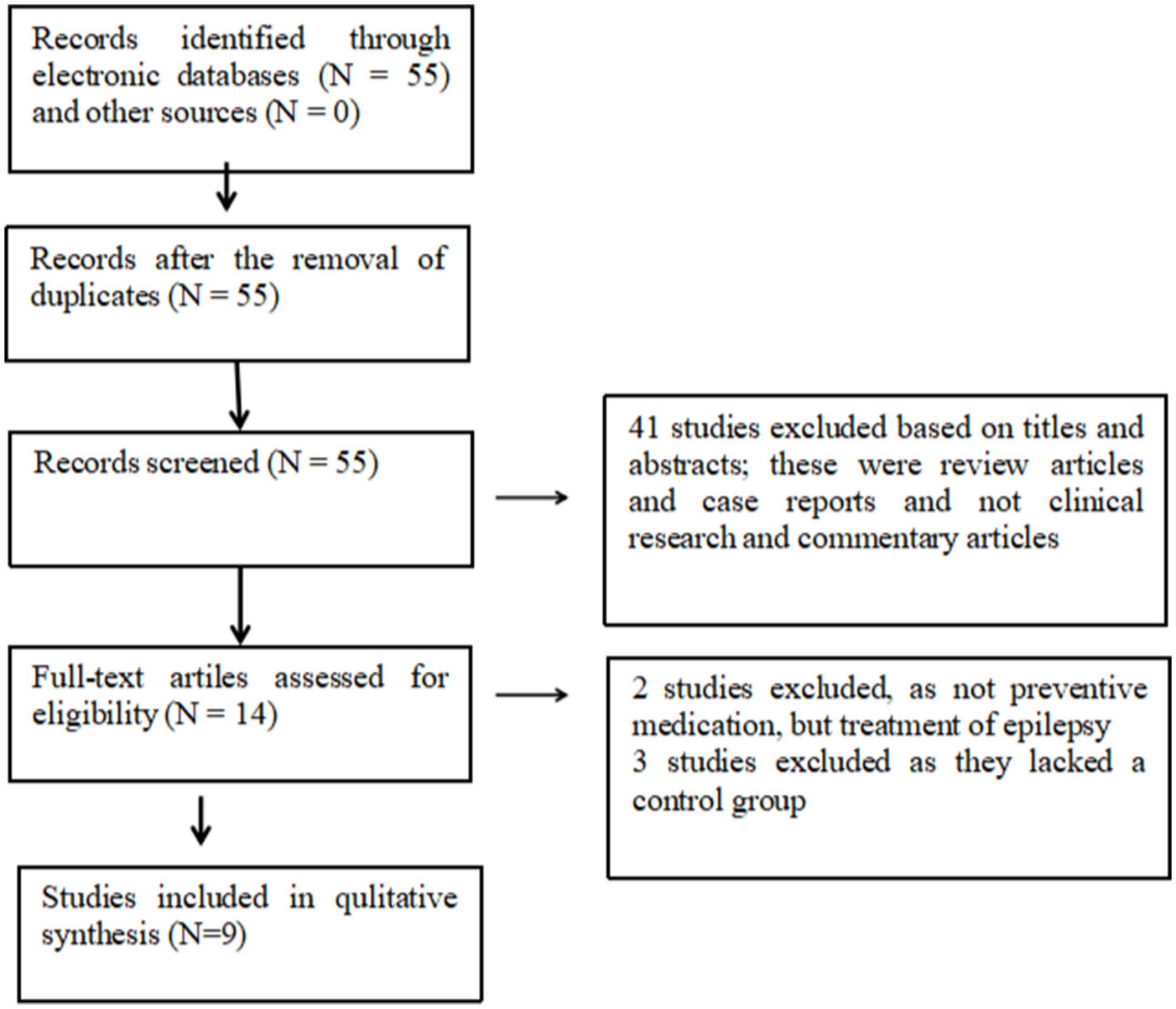
Search strategy for meta-analysis.

In total, 55 relevant papers were initially screened according to the search strategy (Figure 1) and further screened according to the inclusion and exclusion criteria, resulting in the inclusion of 9 relevant studies involving a total of 2,433 patients. Of these, 960 were in the levetiracetam group, 335 were in the no AEDs group, 660 were in the valproate group, and 478 were in the PHT group (Supplementary Table 1).

### 3.2 Literature quality assessment

This review included nine papers, which were all written in the English language, including one RCT and eight cohort studies. The literature reports were all published recently (2008–2023), and the countries studied included the United States, Germany, Canada, Italy, and South Korea. Patients treated with levetiracetam were selected as the experimental group in all studies, and the control group was no ASMs in three cases, PHT in four cases, and VPA in two cases. The NOS methodology was assessed for the nine included studies. The results of the literature quality evaluation are shown in Supplementary Table 2. The quality score of each of the included studies was ≥6, indicating high quality. Egger’s test was applied because fewer than 10 studies were included, with a *p*-value of 0.884 (>0.05) (Supplementary Figure 1). Therefore, no significant publication bias was observed, indicating that the field has a small number of publications.

## 4 Statistical analysis results

### 4.1 Comparison of seizures after prophylactic medication in the levetiracetam and control groups

**Figure 2:**
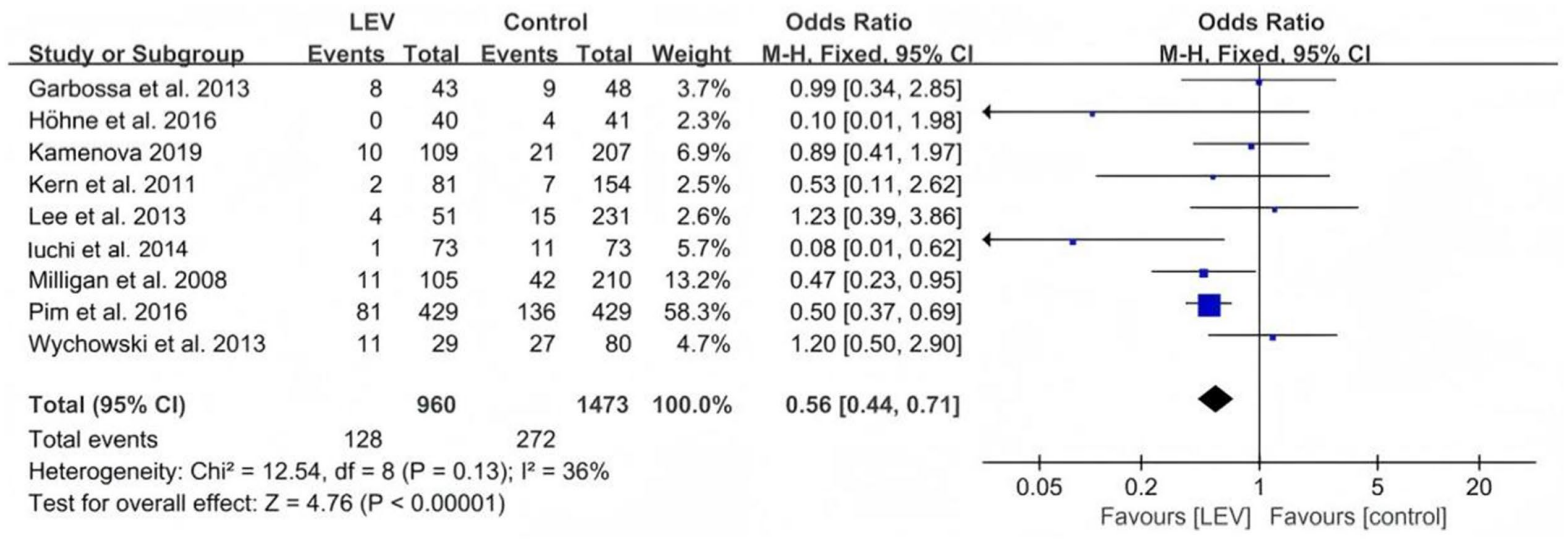
Comparison of the number of seizures in the LEV group and the control group.

Nine studies examined the impact of preventive medication on seizures in patients with brain tumor in both the levetiracetam and control groups. These nine studies were tested for heterogeneity. Homogeneity was observed among the clinical trials (I^2^ = 36.3%, *p* = 0.128), so the fixed-effects model was used for the meta-analysis. The findings indicated a significant difference, with the effectiveness of levetiracetam in preventing seizures being superior to that of the control intervention (odds ratio [OR] = 0.56, 95% confidence interval [CI] 0.44–0.71, *p* < 0.00001, Figure 2).

**Figure 3:**
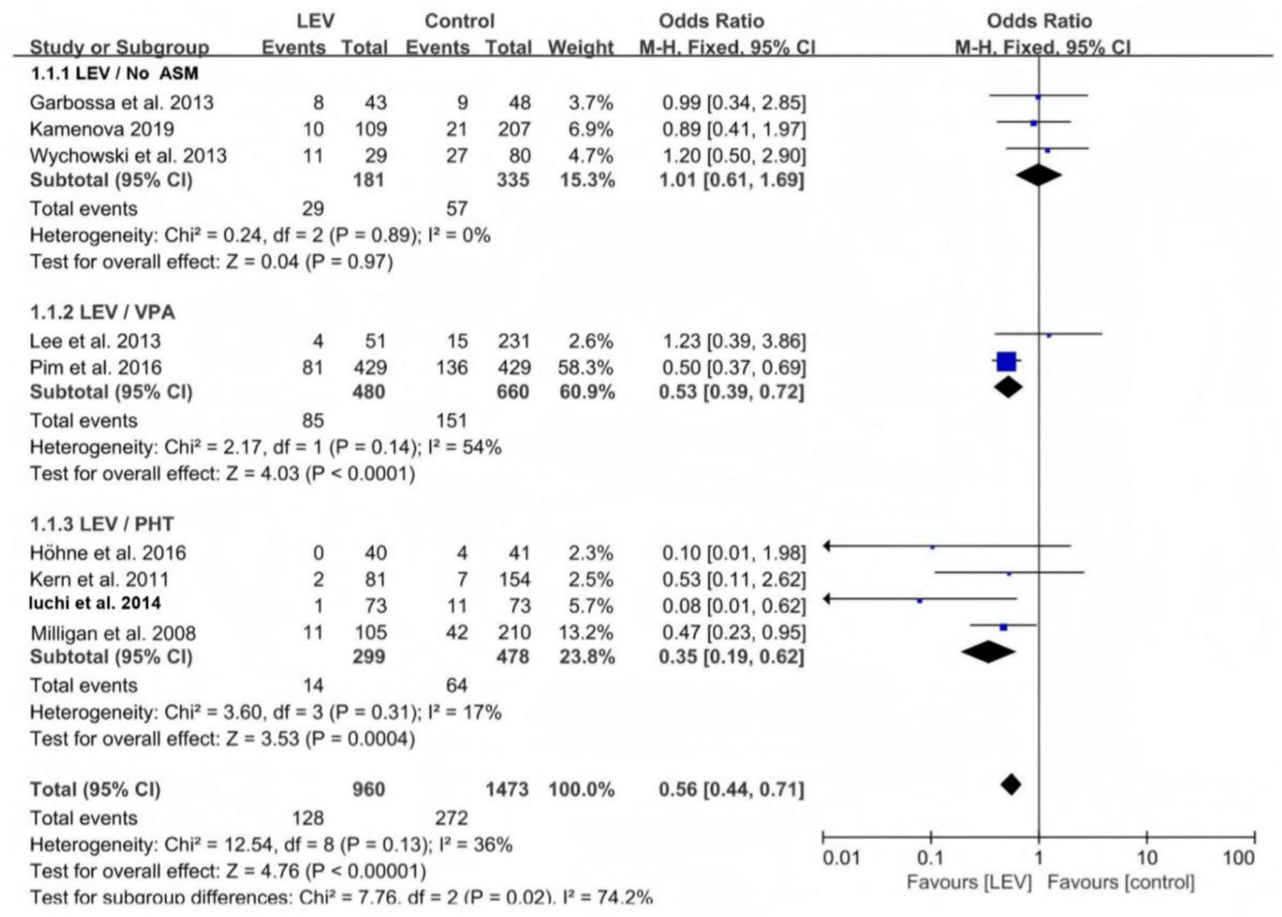
Subgroup analysis of seizures in the levetiracetam and control groups.

**Figure 4:**
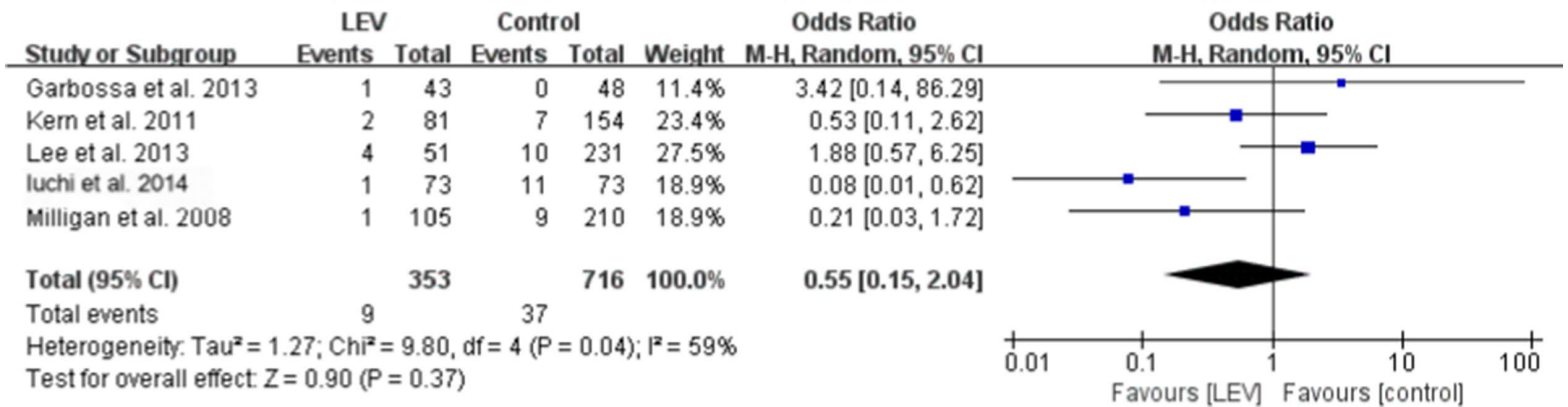
Comparison of early seizures between the levetiracetam and control.

The subgroup analysis comparing the effects of levetiracetam on seizures with no ASMs, valproate, and PHT revealed that the efficacy of levetiracetam was superior to that of valproate (odds ratio [OR] 0.53, 95% CI 0.39–0.72, *p* < 0.0001, Figure 3) and PHT (OR 0.35, 95% CI 0.19–0.62, *p* = 0.0004, Figure 4).

**Figure 5:**
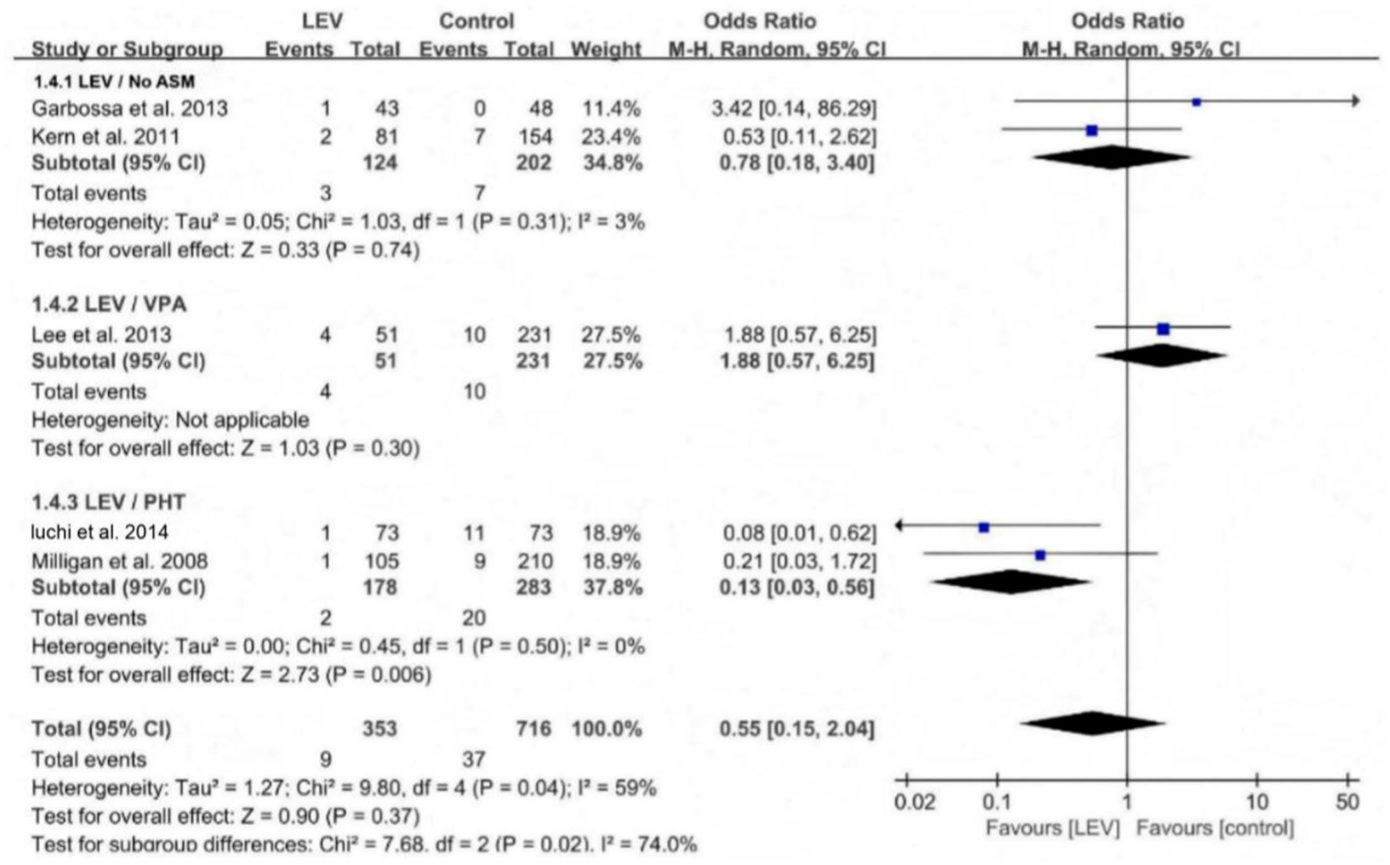
Subgroup analysis of early seizures between the LEV and control groups.

Five studies reported the effect of levetiracetam versus the control intervention on early seizures. These five studies were heterogeneous (I^2^ = 56.3%, *p* = 0.057), so the meta-analysis was performed using the random-effects model. The results revealed that the difference was not statistically significant (OR 0.55, 95% CI 0.15–2.04, *p* = 0.37, Figure 4). A subgroup analysis was performed to explore the sources of heterogeneity. The heterogeneity declined when the different medications used in the control group were divided into three subgroups: No ASMs, VPA, and PHT. The within-group heterogeneity was non-significant in all three subgroups (*p* > 0.1, I^2^ < 50%). The subgroup analysis revealed that the efficacy of levetiracetam was better than that of PHT for preventing early seizures (OR 0.13, 95% CI 0.03–0.56, *p* = 0.006, Figure 5). Only one study included a VPA group; therefore, a subgroup comparison was not possible.

Two studies reported the effect of levetiracetam versus the control intervention on preventing late seizures. These two studies were homogeneous (I^2^ = 0%, *p* = 0.64), so the meta-analysis was performed using the fixed-effects model. The results revealed that the difference was not statistically significant (RR 0.75, 95% CI 0.27–2.03, *p* = 0.57, Supplementary Figure 2).

### 4.2 Comparison of adverse drug reactions between the levetiracetam and control groups

**Figure 6:**
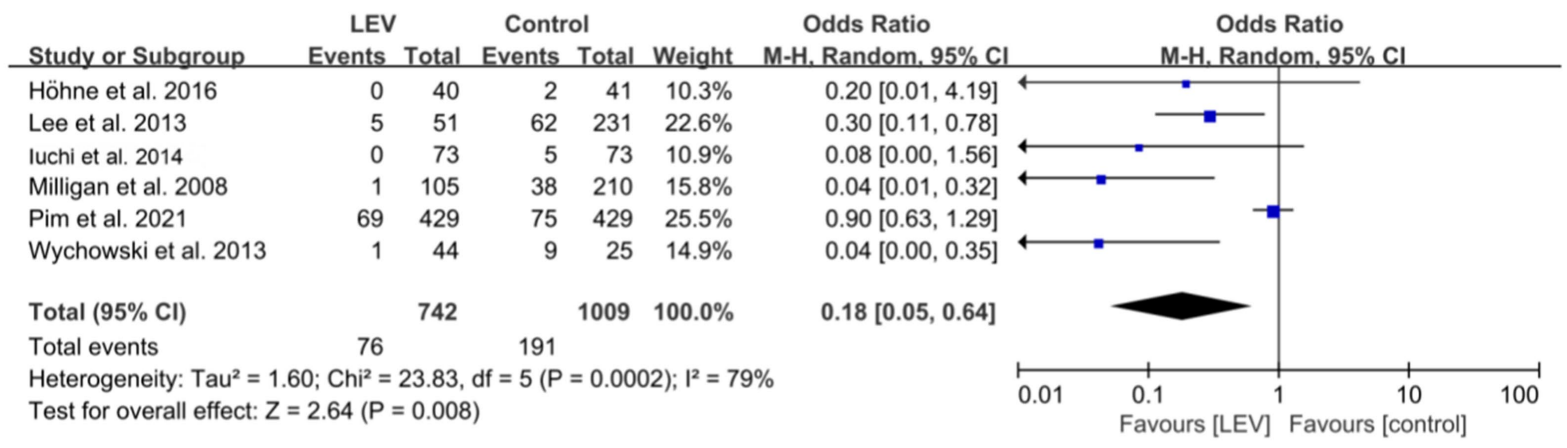
Comparison of adverse drug reactions in the LEV and control groups.

Six studies reported the adverse drug reactions in the two groups of patients, and the heterogeneity test of the above six studies revealed heterogeneity among the clinical trials (I^2^ = 79.1%, *p* = 0.000), so the random-effects model was used for the meta-analysis. The results revealed that the overall difference was statistically significant. Specifically, the incidence of adverse drug reactions was lower in the levetiracetam group than in the control group (OR 0.18, 95% CI 0.05–0.64, *p* = 0.008, Figure 6). Further subgroup analysis revealed that the incidence of adverse reactions with levetiracetam was lower than with PHT (OR 0.06, 95% CI 0.02–0.21, *p* < 0.001, Supplementary Figure 3).

### 4.3 Sensitivity analysis

The stability of the meta-analysis was assessed by sensitivity analysis. When excluding any of the clinical trials, the data from the remaining literature were recombined, resulting in a summary OR of 0.71 (95% CI 0.52–0.98) (Supplementary Figure 4), which was consistent with the results before exclusion, suggesting that the results of the present study were stable.

## 5 Discussion

The use of ASMs in the perioperative period in patients with brain tumor is a topic of debate. Although guidelines from the American Academy of Neurology in 2021 advise against their use, many neurosurgeons around the world still administer ASMs as a standard precaution (5, 7). Patients with brain tumor have a seizure risk of up to 40%, which may be exacerbated by surgery. Perioperative seizures can lead to longer hospital stays, decreased quality of life, reduced survival rates, higher morbidity, and an increased likelihood of developing epilepsy. As a result, some clinicians and neurosurgeons advocate for the use of AEDs as a preventive measure against seizures during the perioperative period (3, 9).

Whether prophylactic levetiracetam reduces the rate of seizures in patients with brain tumor is a matter of controversy (3, 9, 10). A meta-analysis was conducted in 2016 by Pourzitaki; however, the study only included three pre-2014 publications (11). Therefore, clinical practice and data from the last 15 years need to be analyzed in an attempt to provide a basis for resolving the controversy and reaching academic consensus.

An encouraging and positive conclusion obtained from the present study is that prophylactic application of levetiracetam reduces the incidence of epileptic episodes, mainly early seizures, in patients with brain tumor, with superior efficacy to PHT and valproate. Concurrently, levetiracetam results in mild adverse drug reactions; however, the incidence of adverse reactions with levetiracetam is lower than that with PHT and valproate. This is the best evidence to date to guide clinical decision-making, but the findings must be interpreted with caution. Compared with previous meta-analyses (7, 11), we reached a positive conclusion that levetiracetam is effective and safe in preventing epileptic seizures. The meta-analysis by Pourzitaki et al. included only three studies and reported an ambiguous conclusion that the preventive efficacy of levetiracetam seems to be superior to that of PHT and sodium valproate (11). A systematic review conducted by Vyshak et al. concluded that levetiracetam does not reduce epileptic seizures (10). Our study population was comprehensive, with a total of 2,433 patients. Nine publications were included in the meta-analysis, and control populations of patients without AEDs, with PHT, and with valproate were included.

Among the included studies, two retrospective studies compared the administration of LEV with VPA (12, 13). The meta-analysis showed that LEV was superior to VPA in brain tumor seizure prophylaxis. A large-sample study by Pim B et al. (12) showed that LEV was superior to VPA in brain tumor seizure prophylaxis. However, a small-sample study by Lee et al. (13) did not find superiority for LEV. Lee analyzed two factors that led to the negative results, including differences in the preoperative seizure rate between the two groups and the number of patients treated with valproic acid was not well distributed, resulting in a limited indication for the administration of LEV (13). Currently, there is a lack of a definitive explanation for the superiority of LEV over VPA. One possible explanation is that due to the narrow therapeutic index of valproic acid and the unpredictable relationship between its dosage and serum concentration, physicians do not increase the dose of valproic acid sufficiently when it is administered, resulting in the failure of valproate to effectively control epilepsy. Comparatively, LEV has a wider therapeutic index (the ratio between the median toxic dose and the median effective dose), and therefore LEV can be used at higher doses without side effects (12, 14).

Among the included studies, four compared the administration of LEV with PHT (15, 16, 17, 18). The studies conducted by Milligan TA et al. (16), Iuchi et al. (17), and Kern T et al. (18) provide compelling evidence that the prophylactic use of LEV may be more effective for preventing seizure following craniotomy compared to PHT. Our meta-analysis showed that LEV was superior to PHT in brain tumor epilepsy prophylaxis. Several factors need to be considered when interpreting these results. First, the type of pathology in the study population. Among all brain tumors, gliomas exhibit the highest risk of developing epilepsy, while meningiomas present a comparatively lower risk (4). If the study population includes a high percentage of patients with meningiomas, the number of postoperative epileptic events may be limited, thereby adversely affecting the ability to perform statistical analyses. In a randomized controlled trial conducted by Morteza (8), 80 patients with supratentorial brain tumors who underwent craniotomy were randomly assigned to either the LEV group or the PHT group, with 40 patients in each group. Notably, over 65% of the patients in both groups had meningiomas. Throughout the study, a total of 2 seizures were reported in this population: 1 (2.5%) in the phenytoin group and 1 (2.6%) in the levetiracetam group. In contrast, Iuchi’s study reported that the percentage of patients with meningiomas was less than 5%, while the percentage of patients with gliomas exceeded 60% (17). Second, the narrow therapeutic window and nonlinear pharmacokinetic properties of phenytoin sodium mean that even minor adjustments in dosage can cause significant fluctuations in blood concentrations, potentially leading to toxicity and reduced patient compliance (16). In contrast, levetiracetam does not undergo hepatic metabolism and possesses a wide therapeutic index, making it a safer alternative with minimal side effects, even at elevated therapeutic doses (12). Thirdly, PHT may result in significant side effects, such as cardiac events, a reduction in platelet counts, and coagulation abnormalities (18). Recently, Morteza reported that 12.5% of patients in the phenytoin group experienced wound hematomas, 7.5% developed skin rashes, and 2.5% exhibited thrombocytopenia (8).

Our meta-analysis demonstrated that the administration of levetiracetam effectively prevents postoperative seizures. However, subgroup analyses revealed no significant difference between the levetiracetam group and none ASMs group. This finding may seem contradictory and challenging to interpret. In the three studies included in the analysis (19, 20, 21), the control group did not receive any antiepileptic medications, and the incidence of seizures was not elevated. The authors of these studies recognized the limitations of their research and concurred that the observed results might be attributed to the lower prophylactic dose of levetiracetam (500 mg twice a day), rather than the higher doses typically utilized in acute treatment settings. Furthermore, Thomas et al. provided evidence that prophylactic levetiracetam was effective in reducing the incidence of postoperative status epilepticus (21).

Several studies have indicated a lower incidence of epilepsy in the levetiracetam group compared to the control group; however, this difference did not reach statistical significance (15, 16, 22). The researchers also examined the biases and limitations inherent in their studies, noting that these factors may have compromised the accuracy of the results, thereby contributing to the lack of statistical significance. Notably, many researchers failed to establish clear criteria for identifying seizures and relied solely on retrospective analyses of medical records to ascertain the presence or absence of seizures. Additionally, seizures can present atypically, for instance, temporal lobe tumors may manifest as disorientation, while occipital lobe tumors can lead to hallucinations. Such atypical presentations might go unnoticed and unrecorded in medical documentation, potentially leading to an underestimation of the overall incidence of epilepsy (23). Epileptic seizures should be assessed based on reports from patients, family members, and physicians. It remains uncertain whether a daily dose of 1,000 mg of levetiracetam is the optimal prophylactic dosage during the perioperative period. Higher doses may offer improved seizure control. In the control group, the lower doses of ASMs do not achieve effective blood concentrations, and insufficient dosing may result in reduced efficacy (15).

Several studies have shown that levetiracetam is effective in reducing seizures. However, it is important to interpret these results cautiously, as confounding factors such as tumor decortication or total resection, as well as reductions in epileptic electrical activity over time, may influence the perceived effectiveness of levetiracetam (24). In an RCT by Iuchi et al. (17), levetiracetam was effective when compared to PHT. The authors assigned 146 patients to the levetiracetam and PHT groups, with 73 patients in each group. The incidence of early epilepsy was significantly lower in the levetiracetam group, demonstrating its superiority over PHT. However, the conclusions of that study should be interpreted with caution due to limitations in the sample size and result frequency. More recently, Fuller et al. (25) compared the efficacy and safety of levetiracetam with PHT in a randomized prospective study. They revealed a significantly lower incidence of perioperative epilepsy in the levetiracetam group than in the PHT group.

Five studies examined the effectiveness of levetiracetam in preventing early seizures (13, 16–19). Among them, three did not perform statistical analyses (13, 16–17), while two papers did perform statistical analyses but did not show statistical significance (18, 19). The authors noted the very low incidence of epilepsy. Through our meta-analysis of these studies, we demonstrated the efficacy of prophylactic levetiracetam in early seizure prevention. Differences in the efficacy of prophylactic application of levetiracetam in early and late seizures may be related to the different pathogenesis. Early seizures are associated with acute neuronal injury, disruption of the blood–brain barrier, ion channel dysfunction, and electrical excitability abnormalities. In contrast, late seizures are due to glial cell scarring, neurodegeneration, persistent inflammation, and altered synaptic plasticity (4, 26).

The dose, duration, and route of administration of prophylactic levetiracetam lack uniformity. Several prospective studies have demonstrated that prophylactic application of levetiracetam at 1000 mg/day for one week is effective (16, 17). In terms of brain tumor types, the majority of subjects included in the study were gliomas, comprising a total of 720 cases (75.0%). This finding suggests that the use of levetiracetam during the perioperative period for the prevention of early epilepsy in patients with brain gliomas may be of significant importance. The effectiveness of PHT administered after craniocerebral trauma in preventing early seizures has been widely recognized internationally (27). Temkin et al. conducted a comprehensive analysis of six controlled trials and found that preventive treatment using PHT decreased the likelihood of early seizures by 40–50% in patients with craniocerebral injury who underwent craniotomy (28). However, the studies revealed more adverse effects of sodium phenytoin, correlative effects with other drugs, and the need for regular testing of blood levels. Several recent papers have further increased the perception that LEV should be the initial AED consideration in the majority of patients with Brain Tumor-Related Epilepsy (4, 26).

The results of this study revealed that levetiracetam is superior to traditional ASMs, such as sodium valproate and PHT, in terms of adverse drug reactions. Nine studies compared the number of adverse drug reactions between two groups receiving prophylactic medications, showing statistically significant differences. Historically, first-generation ASMs, such as PHT, carbamazepine, and valproate, were commonly prescribed. However, there is strong evidence indicating that the use of traditional ASMs can result in significant adverse effects and interfere with cancer treatments and anesthesia metabolism (29, 30). Levetiracetam is widely used for preventing seizures in patients with craniocerebral injury and cerebrovascular disease because of its good pharmacokinetics and low adverse drug reactions. Levetiracetam exhibits minimal drug interactions and outperforms traditional ASMs in terms of pharmacokinetics, tolerability, safety, and drug interactions (5, 30). Close monitoring by continuous blood collection is not required because the therapeutic index is broader and considers its potential synergistic effect on tumor therapy. This reduces the therapeutic risk for patients with severe and complex diseases that require multi-drug combinations, especially patients with brain tumor requiring a combination of chemotherapy, radiotherapy, and surgery. According to the findings of Konrath et al. (31) and Rahman et al. (32), the preventative use of levetiracetam did not result in any negative impact on cognitive ability, quality of life, or hematologic issues among individuals with brain tumor.

### 5.1 Study limitations

This meta-analysis has some limitations that should be considered. The included studies were of poor quality, each posing a high risk of bias. Our meta-analysis of RCTs was limited in scope, leading us to incorporate observational studies to broaden our analysis. The inclusion of these studies may have introduced several confounding factors, such as differences in baseline information between the study groups, as well as different drug doses, medication durations, and timing of endpoint events. To establish a more reliable and objective evidence base, future large-sample and ethnically diverse RCTs should be conducted collaboratively across multiple medical centers. Additionally, the proportion of brain tumor types varied among the study populations, with some studies focusing on all brain tumors (including benign and malignant ones such as gliomas and metastatic tumors) and others only studying gliomas. Further investigation is needed to understand the true differences among brain tumor subgroups given the relatively low incidence of epileptic events.

## 6 Conclusion

Our results suggest that the prophylactic application of levetiracetam reduces the incidence of epilepsy, specifically early epilepsy, in patients with brain tumor, with superior efficacy to PHT and valproate. Furthermore, levetiracetam is associated with mild adverse drug reactions, and the incidence of adverse effects with levetiracetam is lower than that with PHT and valproate. Consensus and clinical practice guidelines should consider the evidence presented in this meta-analysis to guide future decisions. However, the literature has a risk of bias. Therefore, joint large-sample RCTs from multiple medical centers are required to confirm the superiority of the prophylactic application of levetiracetam in controlling perioperative epileptic seizures.

## Data Availability

The raw data supporting the conclusions of this article will be made available by the authors, without undue reservation.

## Supplementary material

SUPPLEMENTARY FIGURE 1 Egger’s test for the included studies.

SUPPLEMENTARY FIGURE 2 Comparison of late seizures in the LEV and control groups.

SUPPLEMENTARY FIGURE 3 Subgroup analysis of adverse drug reactions in the LEV and control groups.

SUPPLEMENTARY FIGURE 4 Sensitivity analysis of the included studies.
