# Supplementary figures and images for: Efficacy and safety of levetiracetam in preventing postoperative seizures in adult patients with brain tumors: a meta-analysis
Source: Front Neurol. 2025 Mar 7;16:1543905. doi: 10.3389/fneur.2025.1543905 (PMC11925779; doi:10.3389/fneur.2025.1543905)

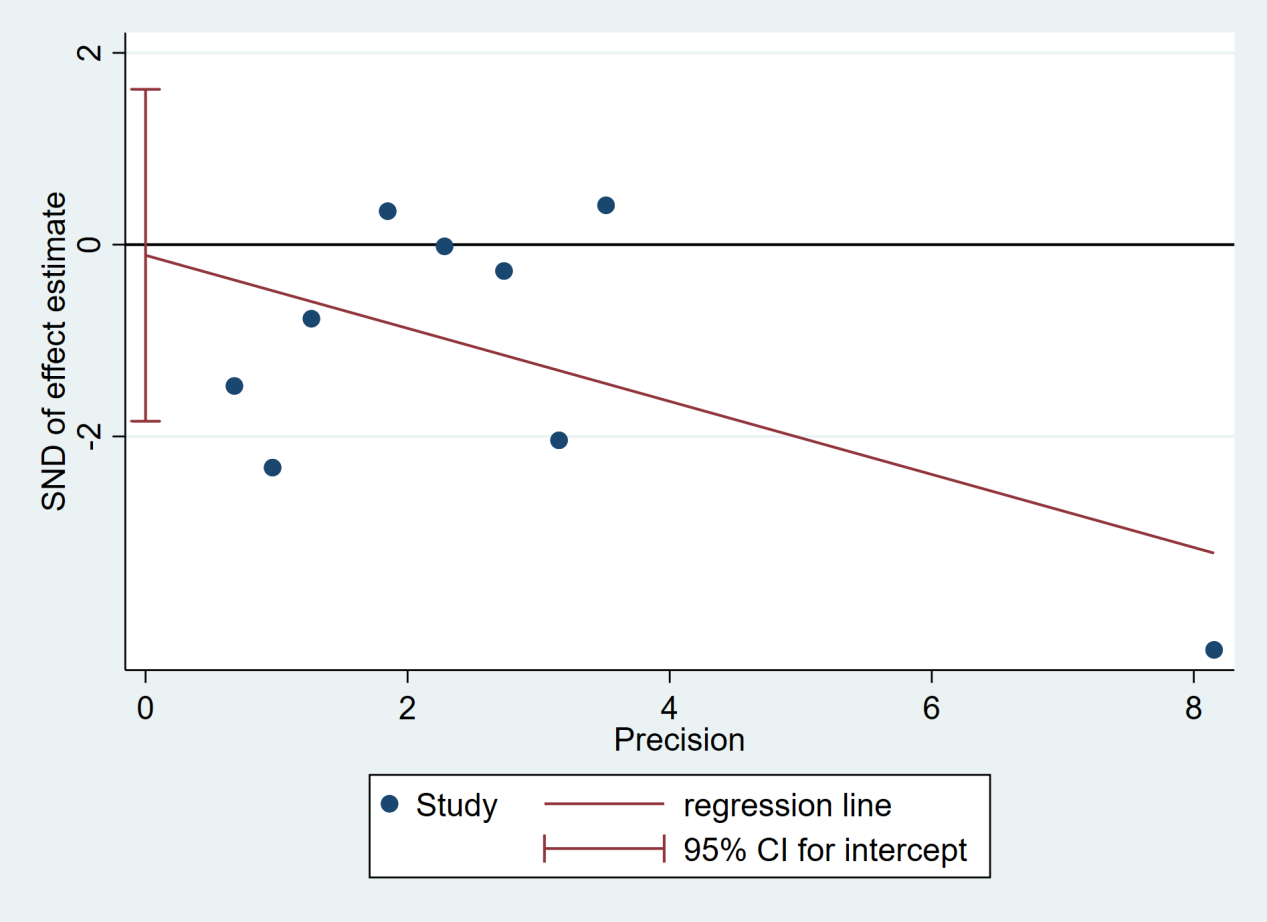

Supplement: SUPPLEMENTARY FIGURE 1 — Egger’s test for the included studies. [file Image_1.tif]

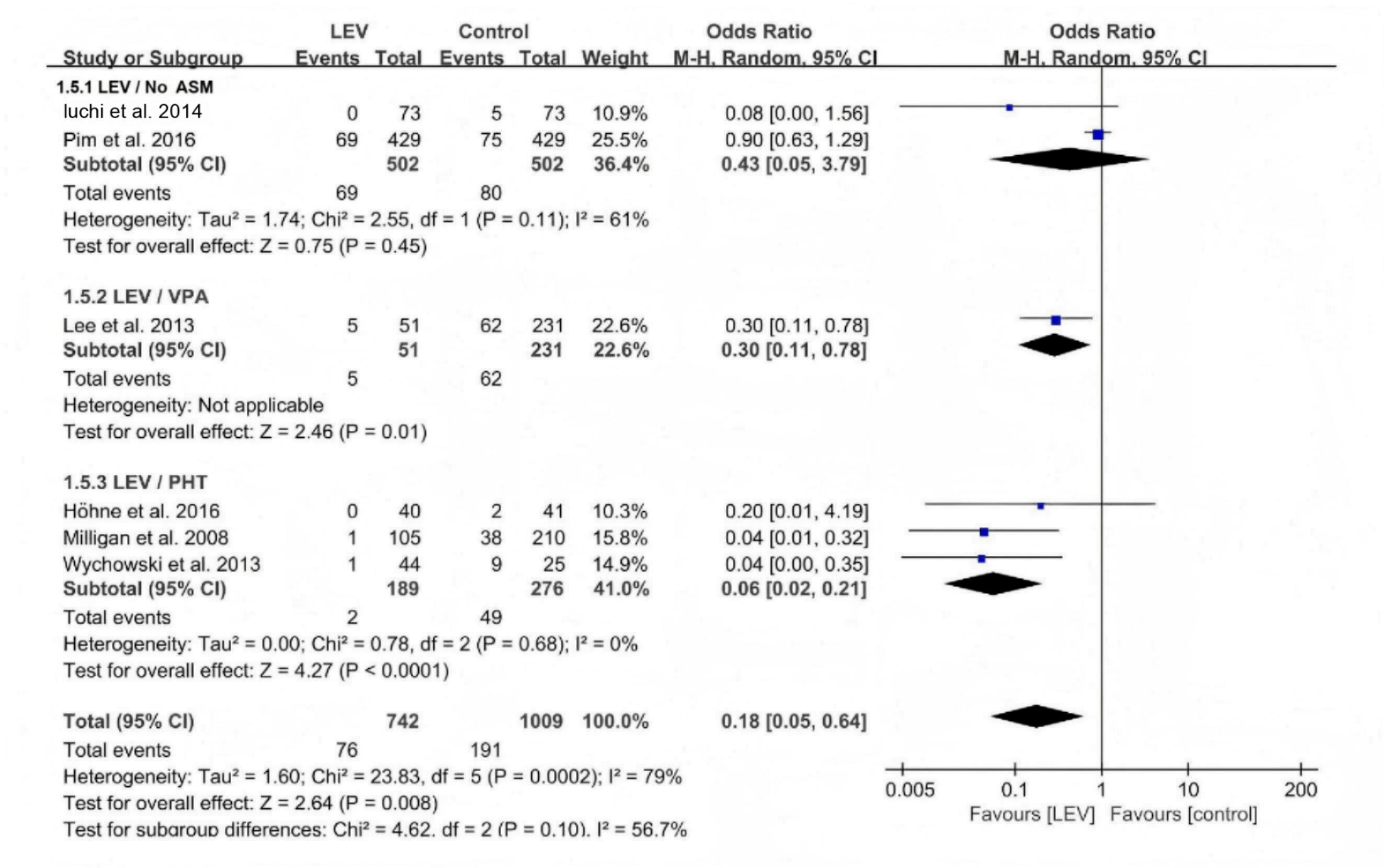

Supplement: SUPPLEMENTARY FIGURE 3 — Subgroup analysis of adverse drug reactions in the LEV and control groups. [file Image_3.jpeg]

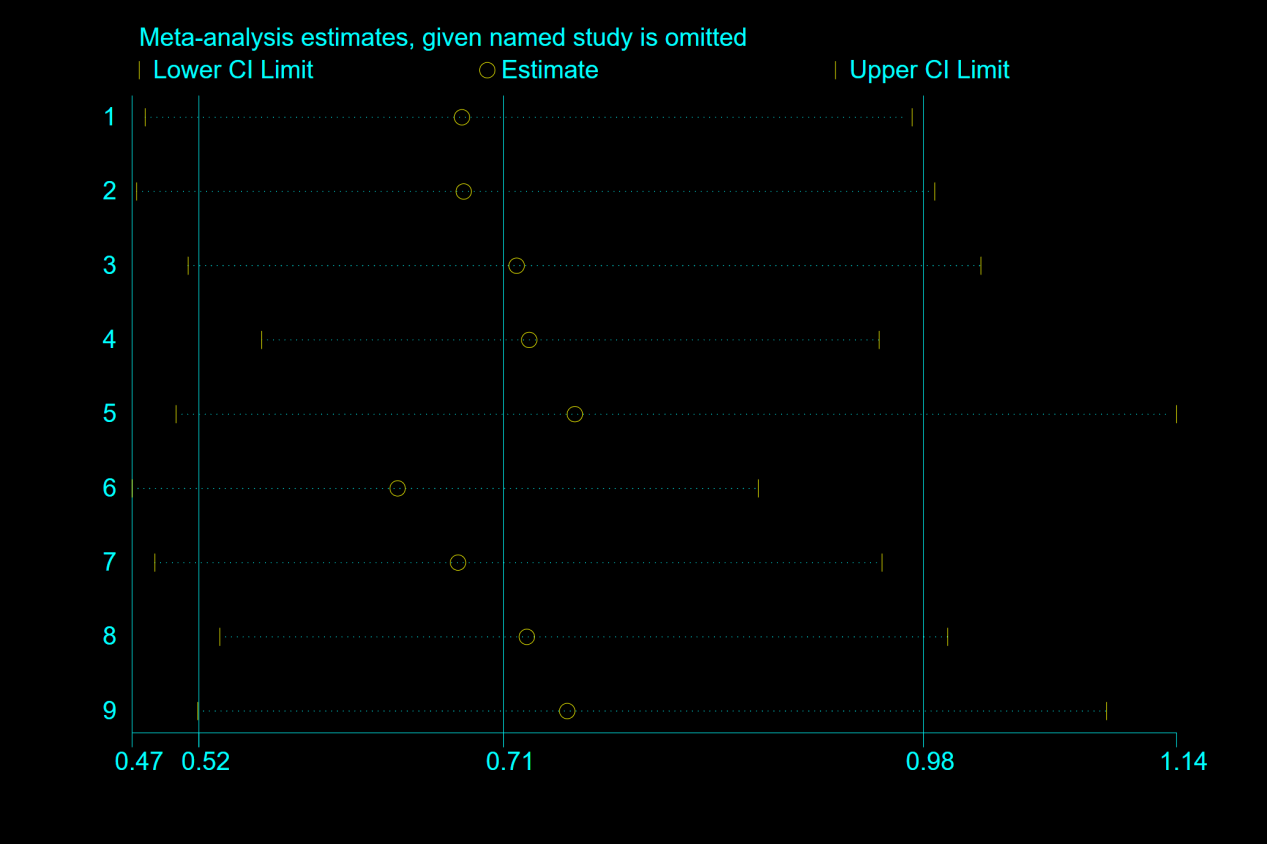

Supplement: SUPPLEMENTARY FIGURE 4 — Sensitivity analysis of the included studies. [file Image_4.tif]
