# Supplementary material for: Efficacy and safety of levetiracetam in preventing postoperative seizures in adult patients with brain tumors: a meta-analysis
Source: Front Neurol. 2025 Mar 7;16:1543905. doi: 10.3389/fneur.2025.1543905 (PMC11925779; doi:10.3389/fneur.2025.1543905)
Supplement: Supplementary file 5 [file Table_1.docx]

**Supplementary Table 1 Basic information of the included literature**

| **First author and year of publication** | **Country** | **Research target** | **LEV group** | | | **Control subjects** | | **Statisticians** | |
| --- | --- | --- | --- | --- | --- | --- | --- | --- | --- |
|  |  |  | **Number** | **Intervention dose** | **Follow-up time** | **Number of examples** | **Intervention dose** | **Efficacy** | **Safety** |
| Garbossa D et al., 2013[19] | Italy | glioblastoma | 43 | Start 3–5 days preoperatively, 1000 mg/day for 6 months | 6 months | No AED 48 | not mentioned | pointless | Statistically insignificant |
| Kamenova M et al., 2020[20] | Palestine | supratentorial brain tumor | 109 | 500–3000 mg/day for 2 weeks-January | 332 (±332.6) days | No AED 207 | not mentioned | pointless | Statistically insignificant |
| Kern K et al., 2012[18] | German | supratentorial brain tumor | 81 | 2000–500 mg/day for 5 days | 7 days | PHT 154 | 300–50 mg/day | pointless | Low number of adverse reactions, not statistically treated |
| Iuchi T et al., 2015[17] | Japanese | supratentorial brain tumor | 73 | 1000 mg/day for 7 days | 7 days | PHT 73 | Starting dose 15–18 mg/kg, maintenance 250 mg/day | have significance | Low number of adverse reactions, not statistically treated |
| Pim B et al., 2021[12] | The Netherlands | glioma | 429 | 2000 mg/day | 36 months | VPA 429 | 1500 mg/day | levetiracetam superior to sodium valproate | Not statistically significant |
| Wychowski T et al., 2013[21] | USA | glioblastoma | 29 | 1000 mg/day | 36 months | No AED 80 | not mentioned | pointless | Low number of adverse reactions, not statistically treated |
| Lee YJ et al.,2013[13] | South Korea | supratentorial brain tumor | 51 | 500 mg q12h IV, 1000 mg/day for 1 month | 16.8 ± 9.9 months | VPA 231 | 600mg q12h IV, 1600 mg/day | pointless | Statistically significant |
| Hohne J et al., 2016[15] | German | supratentorial brain tumor | 40 | 1000–3000 mg for 5 days | In-hospital | PHT 41 | Loading dose 15–20 mg/kg | Favorable, not statistically significant | Adverse effects were mild and not statistically significant. |
| Milligan TA, 2008[16] | USA | supratentorial brain tumor | 105 | 1000 mg for 1 week | 1 year | PHT 210 | 300–1000 mg | Favorable, not statistically significant | Statistically significant |
