# Supplementary material for: Efficacy and safety of levetiracetam in preventing postoperative seizures in adult patients with brain tumors: a meta-analysis
Source: Front Neurol. 2025 Mar 7;16:1543905. doi: 10.3389/fneur.2025.1543905 (PMC11925779; doi:10.3389/fneur.2025.1543905)
Supplement: Supplementary file 6 [file Table_2.docx]

**Supplementary Table 2 Quality evaluation of the included literature (NOS scale)**

| **First author and years of publication** | **Type of study** | **Option** | **Comparative** | **Follow-up** |
| --- | --- | --- | --- | --- |
| Garbossa D et al., 2013 | Retrospective | 4 | 2 | 3 |
| Kamenova M et al., 2019 | Cohort study | 4 | 2 | 3 |
| Kern K et al., 2012 | Cohort study | 3 | 2 | 2 |
| Iuchi T et al., 2015 | Retrospective | 4 | 2 | 3 |
| Pim B et al., 2021 | Cohort study | 3 | 2 | 3 |
| Wychowski T et al., 2013 | Randomized controlled | 3 | 1 | 2 |
| Lee YJ et al., 2013 | Retrospective | 4 | 2 | 3 |
| Hohne J et al., 2016 | Observational studies | 3 | 1 | 3 |
| Milligan TA, 2008 | Retrospective | 3 | 3 | 3 |
